# Immunoexpression of lactoferrin in bone metastases and corresponding primary carcinomas

**DOI:** 10.3892/ol.2013.1227

**Published:** 2013-03-05

**Authors:** A. IENI, V. BARRESI, G. BRANCA, G. GIUFFRÈ, M.A. ROSA, G. TUCCARI

**Affiliations:** 1 Department of Human Pathology, Section of Pathological Anatomy, University of Messina, Messina, Italy;; 2 Department of Surgical Specialties, Section of Oncological Orthopaedics, University of Messina, Messina, Italy

**Keywords:** immunohistochemistry, lactoferrin, bone tissue, metastasis, carcinoma

## Abstract

Although the immunohistochemical presence of lactoferrin (LF) in pathological neoplastic bone and cartilage samples has previously been studied, no data concerning the distribution of LF in bone metastases of cancers that have originated from different organs are available at present. Consequently, using a monoclonal antibody, we have investigated the immunohistochemical LF pattern in 50 formalin-fixed and paraffin-embedded samples of human bone metastases and their corresponding primary carcinoma tumours (breast, 8; prostate, 4; kidney, 4; lung, 3; colon-rectum, 2 and uterus, 4). Quantification of LF immunoreactivity was performed using an intensity distribution (ID) score. LF immuno staining with a variable ID score was encountered in 11/25 (44%) metastatic lesions. In particular, the LF immunoreactivity was identified with a percentage ranging from 50 to 75% of bone metastases due to prostatic, renal, uterine and colorectal carcinomas; the positivity decreased in breast carcinomas (37.5%) and was completely absent in lung cancers. No differences in the LF-ID score were observed between primary and metastatic neoplastic localisations. Additionally, no correlations were identified between LF immunoexpression and the other parameters tested, including the age and gender of patients. Regardless of the mechanism of action of LF in human malignant tumours, we identified LF immunohistochemical reproducibility at primary and metastatic sites. Therefore, we hypothesise that the presence of LF in native neoplastic carcinomatous clones is maintained in secondary bone metastatic deposits.

## Introduction

Lactoferrin (LF) is a single-chain non-haeme iron-binding glycoprotein with a molecular weight of ∼80 kDa, consisting of ∼700 amino acids with a high degree of homology between species (1). The concentration of LF is high in milk, mainly in colostrum, although it has been found in other body fluids and secretions such as blood plasma, tears, saliva, urine, bile, semen and amniotic fluid (2–4).

LF has a wide range of biological activities, including antimicrobial properties, improvement of iron status, anti-inflammation, development of immune function and promotion of cell proliferation during carcinogenesis (5,6).

Using immunohistochemistry, the distribution of LF has been investigated in normal human fetal and adult tissues including the stomach, kidney, lung, pancreas, liver, bone marrow and skin (7,8). More recently, the immunohistochemical distribution of LF has been analysed in human embryonic, fetal and adult bone and cartilaginous tissues (9), in order to investigate whether LF may be involved in the growth and differentiation of the human skeleton, similar to that suggested in murine models as well as in cell culture lines (10–12). In addition, our research group has also evaluated the immuno histochemical presence of LF in pathological neoplastic bone and cartilage samples (13,14). LF immunoreactivity was revealed in chondroblastomas, chondromyxoid fibromas, giant cell tumours, osteoid osteomas, myelomas and adamantinomas; while no LF immunoexpression was detected in enchondromas, osteochondromas, ossifying fibromas, chondrosarcomas or osteosarcomas (13–15).

As no data regarding the distribution of LF in bone metastases of cancers that have originated from different organs are available at present, we set out to analyse the immunohistochemical pattern of LF in a cohort of these samples as well as in the corresponding primary neoplasms using a monoclonal antibody against LF.

## Materials and methods

### Specimens

LF immunoexpression was investigated in 25 specimens of human bone metastatic lesions obtained through curettage or surgery from an equal number of patients (16 females, 9 males; mean ages, 64 and 92 years, respectively; age range, 28–85 years). Data concerning the site of occurrence of the metastases as well as surgical samples of the primary corresponding carcinomas were obtained from the files at the Department of Human Pathology, University of Messina, Messina, Italy. The primary carcinoma sites included breast (8 cases), prostate (4 cases), kidney (4 cases), lung (3 cases), colon-rectum (2 cases) and uterus (4 cases).

### Preparation of specimens

All samples were fixed in 10% neutral formalin for 24–36 h at room temperature (RT), and then embedded in paraffin at 56°C. The bone metastatic specimens were subjected to a decalcification procedure performed using formic acid (5%) or ethylenediamine-tetraacetic acid (EDTA; 5%, pH 7.4) for ≤12–24 h, depending on the size of mineralised samples. From each tissue block, 4-*μ*m sections were stained with haematoxylin and eosin (H&E) for microscopic evaluation. Parallel sections were cut and mounted on silane-coated glass, then dewaxed in xylene and rehydrated in graded ethanols. Antigen retrieval was performed prior to the addition of the primary antibody, by heating slides placed in 0.01 M citrate buffer at pH 6.0 in a microwave oven (750 W) for three 5-min cycles.

### Immunohistochemistry

For the immunohistochemical study, sections were treated in a moist chamber with: i) 0.1% H_2_O_2_ in methanol for 30 min at RT, to block the intrinsic peroxidase activity; ii) normal sheep serum to prevent non-specific adherence of serum proteins; iii) mouse monoclonal primary antibody (anti-human) against LF [clone 1A1; working dilution (wd), 1:75; Biodesign International, Inc., Saco, ME, USA] for 60 min at RT; iv) sheep anti-mouse immunoglobulin antiserum (wd, 1:25; Behring Institute, Marburg, Germany) for 30 min at RT; v) mouse anti-horseradish peroxidase-antiperoxidase complexes (wd, 1:25; DakoCytomation, Copenhagen, Denmark) for 30 min at RT. To reveal peroxidase activity, the sections were incubated in the dark for 10 min with 100 mg 3,3′-diaminobenzidine tetrahydrochloride (Sigma, St. Louis, MO, USA) in 200 ml 0.03% hydrogen peroxide in phosphate-buffered saline (PBS) solution. The nuclear counterstaining was performed using Mayer’s haemalum solution.

Renal tubular structures within normal kidney samples and portions of the parotid gland were utilised as LF-positive controls. In addition, the LF immunoreactivity demonstrated in granules of polymorphonuclear neutrophils inside the neoplastic lesions was utilised as additional positive control. Finally, to test the inter-run variability of LF immunostaining, the same LF-positive parotid sample was utilised in every run. To test the of LF immunoreaction in order to omit the possibility of non-specific reaction, serial sections of each affected specimen were tested by replacing the specific antiserum with either PBS, normal rabbit serum, or by absorption with an excess of purified human LF from human liver and spleen (Sigma) as well as with pre-absorbed primary antibody; the results obtained were negative.

### Microscopy

The analysis of immunostained sections was estimated by light micro scopy using ×20 and ×40 objective lenses and a ×10 eyepiece. Two pathologists used a double-headed microscope to perform the assessment of LF immunostained sections on a consensus basis. The percentage of stained neoplastic cells (area of staining positivity, ASP) was graded as follows: 0, no staining; 1, >0–5%; 2, >5–50% and 3, >50%. The intensity of staining (IS; weak, 1; moderate, 2; strong, 3) was also assessed. Then an LF intensity distribution (ID) score was calculated for each case by multiplying the values of the ASP and the IS, according to that described by Tuccari *et al*(16).

### Statistical analysis

The correlations between LF immuno-expression and the clinical data (age and gender of patients and site of the lesion) were investigated using either the χ^2^ or the Fisher’s exact test, as appropriate. Moreover, the correlation between the LF immunoreactivity pattern in primary carcinomas and the corresponding metastatic bone samples was analysed by a Spearman’s rank correlation test. P<0.05 was considered to indicate a statistically significant difference. Data were analysed using the Statistical Package for the Social Sciences (SPSS) software, version 6.1.3 (SPSS, Inc., Chicago, IL, USA).

## Results

Routinely stained H&E sections exhibited a good morphology, confirming the histopathological diagnosis of all cases, either in the primary neoplastic lesions or in the bone neoplastic deposits. Clinicopathological and immunohistochemical data for LF relative to the 25 analysed bone metastatic samples as well as the corresponding primary carcinomas are listed in Table I.

**Figure 1:**
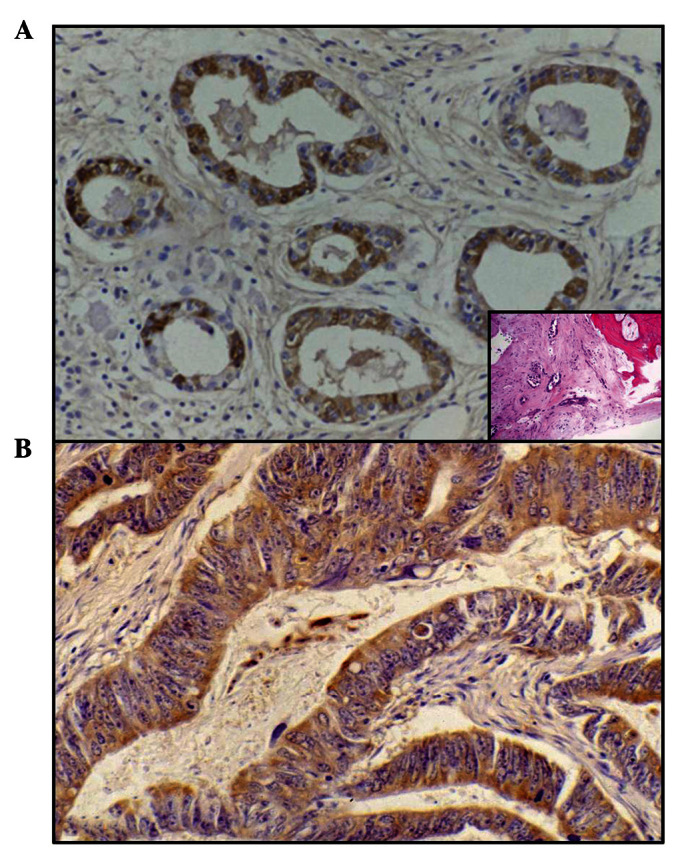
(A) At greater magnification LF immunoreactivity is evident in certain prostatic elements close to others that are unreactive, localised inside the bone tissue (original magnification, ×160). The inset reveals the corresponding haematoxylin and eosin routinely stained section of the bone metastatic site (original magnification, ×40). (B) A diffuse LF immunostaining is demonstrated in the neoplastic uterine glands (original magnification, ×300).

**Figure 2:**
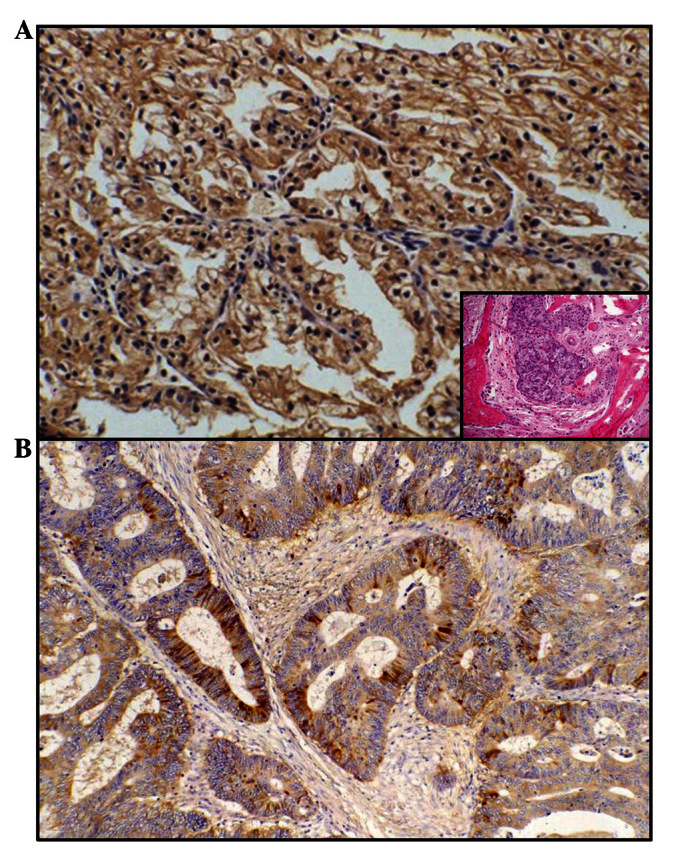
(A) LF cytoplasmic positivity is evident at the periphery of clear cells of renal carcinoma metastatic to bone (original magnification, ×200). The inset illustrates the corresponding haematoxylin and eosin routinely stained section of the bone metastatic site (original magnification, ×40). (B) Immunoreactive colonic cells are in contact with negative cells inside the bone neoplastic deposits (original magnification, ×240).

LF immunostaining with a variable ID score was encountered in 11/25 (44%) metastatic lesions; LF was identified in 7/16 (43.8%) female and 4/9 (44.4%) male patients. In particular, LF immunoreactivity was identified with a percentage ranging from 50 to 75% of the cases of bone metastases due to prostatic (Fig. 1A, inset), uterine (Fig. 1B), renal (Fig. 2A, inset) and colorectal (Fig. 2B) carcinomas (Table I). Additionally, the positivity was decreased in breast carcinomas (37.5%) and was completely absent in lung cancers (Table I). The immunostaining was mainly localised in the cytoplasm of the neoplastic elements and occasionally in the nuclei of the same cells. No differences in LF-ID score were observed between the primary and metastatic neoplastic localisations with an equivalent LF immunoreactivity, either regarding the intensity or the percentage of stained cells.

LF was evident in renal tubular structures, parotid ductular/acinar portions and in granules of polymorphonuclear neutrophils utilised as positive controls.

No correlations were observed between LF immunoexpression and the other parameters investiaged, including the age and gender of the patients and the localisation of neoplastic metastatic lesions in bones.

## Discussion

A number of studies have demonstrated promising results in the potential use of LF for the improvement of bone health (12,17–20). In particular, LF stimulates the proliferation, differentiation and survival of osteoblasts (20), as well as significantly increasing the mineral apposition rate and bone formation, as demonstrated by the assessment of dynamic histomorphometric indices (12). Consequently, it has been suggested that LF may be useful in pathological states of reduced bone density when used either systemically or locally.

As part of a series of studies concerning the immunohistochemical distribution pattern of LF in human neoplasms (21), we have previously investigated this distribution in pathological primary neoplastic bone and cartilage samples, as well as in the corresponding human normal embryo-fetal bone and cartilage tissues (9,13). The observed heterogeneous distribution of LF in tumours, as well as its independence from benign and malignant characteristics, appear to contrast with the elsewhere hypothesised role of LF as oncofetal marker (15). The most aggressive bone tumours, such as osteosarcomas and chondrosarcomas, were consistently observed to be unreactive for LF; while the pattern of LF expression was mainly evident in the early phases of bone growth, suggesting an important role for LF as a bone growth regulator in the early phases of skeletal development, particularly in endochondral ossification (12,15).

Metastatic deposits in bones from carcinomas arising from breast, colon, endometrium, kidney, lung and prostate are considered to be a key stage in the natural history of these neoplasms, although to date no data regarding the immunohistochemical distribution of LF have been available in the literature. In the current study, we immunohistochemically detected a variable ID score for LF in the cytoplasm of 11/25 (44%) metastatic neoplastic bone lesions as well as in the corresponding primary carcinomas. Occasionally, the site of LF immuno staining was appreciable both in the nucleus and cytoplasm, and this co-localisation was expected, as LF has also been revealed in the nucleoli and LF is speculated to be involved in ribosomal biogenesis (21,22). With regard to the site of the primary carcinomas, LF immunoreactivity was found with a percentage ranging from 50 to 75% of bone metastases due to colorectal, uterine, prostatic and renal carcinomas. In addition, the positivity was decreased in breast carcinomas (37.5%) and was completely absent in lung cancers. In these primary neoplastic conditions, previous studies by our research group have demonstrated a similar variable percentage of immuno expression of LF (23–26). In detail, a progressive increase of LF immunostaining was encountered when moving from endometrial adenocarcinomas (61%) (25) and renal cell carcinomas (62.5%) (26) to well-differentiated prostatic adenocarcinomas (66%) (23), and finally to adenocarcinomas and colloid colorectal carcinomas (80%) (24). The most likely explanation for the negative LF immunoreactivity observed in a number of cases of the above mentioned cohorts of tumours has been correlated with undifferentiated or less differentiated variants of carcinomas (23–26). Occasional and slight LF staining has been found in isolated cells of undifferentiated prostatic carcinomas (23), while only well- and moderately differentiated colonic carcinomas exhibited a strong LF reaction. Furthermore, a significantly higher LF-ID score was evident in the endometrioid type in comparison to the non-endometrioid type carcinomas of the uterus (25). In addition, significant differences in the LF-ID score were found among clear cell renal carcinomas (CCC) and other non-CCC variants, the former exhibited a lower score (26). By contrast, the positive rate of LF in breast carcinoma has been identified with a large variability, ranging from 7.5 to 42% of cases (27,28). However, LF was more often observed in low-grade ductal carcinomas with positive estrogen/progesterone receptors, confirming a decrease in LF immunostaining in less differentiated and more aggressive breast carcinomas (27–29). Therefore, LF may be a potential marker for glandular or acinar differentiation, similar to that previously observed in other malignancies (28,30,31). No data concerning LF immunodistribution in primary and metastatic lung cancer are currently available in literature.

The origin of LF in human malignant primary and metastatic tumours has not yet been fully elucidated. It is well known that LF has a high affinity for iron, which is considered to be an essential nutrient for cells that are dividing rapidly, such as tumour cells, taking part in various metabolic processes (including oxydative phosphorylation and RNA/DNA synthesis) (32,33). Therefore, neoplastic elements may produce LF in order to provide a greater amount of iron available for their turnover, as we have previously suggested (21,24,26,34,35). Alternatively, the localisation of LF in malignant cells may not reflect an intracellular synthesis, but rather the degree of transmembranous iron transfer as the consequence of defective or functionally impaired LF-receptors already documented on the surface of target cells as well as in human neoplastic cell lines (36,37). It has been suggested that LF is involved in the regulation of certain important processes, such as the cell cycle and cell death, resistance to carcinogenesis and the development of metastases (26,38). Other potential mechanisms have also been suggested with regard to the role of LF in the process of human carcinogenesis. These include induction of programmed cell death, prevention of angiogenesis and regulation of cell cycle protein expression (39,40). LF is able to trigger the apoptotic process by the activation of caspase-3 and -8 as well as the FAS signaling pathway (41,42). By contrast, LF has also been demonstrated to inhibit tumour-initiated angiogenesis *in vitro* and *in vivo*, possibly by blocking endothelial function and inducing IL-18 production (39,43,44). In addition, it has been demonstrated that LF promoted growth arrest either at the G1-S transition in breast cancer cells (43) as well as at the G0-G1 checkpoint in oral and neck cancer cells (44). However, regardless of the mechanism of action of LF in human malignant tumours, we have identified LF immunohistochemical reproducibility at primary and metastatic sites. Therefore, we hypothesise that the appearance of LF in native neoplastic carcinomatous clones is maintained in secondary bone metastatic deposits. However, additional investigations are required, mainly regarding the potential for new applications of LF in cancer treatment, due to its nutraceutical function and its ability to potentiate chemotherapy.

## Figures and Tables

**Table I:** Clinicopathological and LF immunohistochemical data concerning bone metastases.

Case no.	Gender	Age	Primary site of neoplasms	Histotype of carcinoma	Grading	Site of bone metastases	LF-ASP	LF-IS	LF-ID score
1	F	58	Breast	Medullary	-	Femur	0	0	0
2	M	59	Prostate	Cribriform	G2	Femur	1	2	2
3	F	54	Breast	Ductal invasive	G3	Humerus	0	0	0
4	F	80	Breast	Ductal invasive	G2	Femur	2	1	2
5	F	28	Uterus	Endometrioid	G2	Vertebra	2	2	4
6	F	80	Colon-rectum	Adenocarcinoma	G2	Femur	1	2	2
7	F	55	Breast	Ductal invasive	G2	Femur	1	2	2
8	M	56	Lung	Small cell	-	Sternum	0	0	0
9	F	70	Lung	Adenocarcinoma	G3	Femur	0	0	0
10	F	60	Breast	Lobular invasive	-	Femur	0	0	0
11	M	61	Lung	Small cell	-	Fibula	0	0	0
12	M	74	Kidney	Clear cell	G2	Femur	0	0	0
13	F	69	Uterus	Serous	G3	Humerus	0	0	0
14	F	62	Breast	Ductal invasive	G1	Vertebra	2	2	4
15	F	69	Colon-rectum	Adenocarcinoma	G3	Femur	0	0	0
16	F	76	Uterus	Endometrioid	G2	Femur	1	1	1
17	M	75	Prostate	Undifferentiated	G3	Pelvis	0	0	0
18	F	75	Breast	Ductal invasive	G3	Vertebra	0	0	0
19	M	70	Kidney	Chromophobe	G2	Femur	2	2	4
20	M	85	Prostate	Adenocarcinoma	G1	Vertebra	2	2	4
21	F	58	Kidney	Clear cell	G2	Vertebra	1	1	1
22	M	71	Prostate	Adenocarcinoma	G2	Femur	1	2	2
23	F	52	Breast	Lobular invasive	-	Femur	0	0	0
24	F	67	Uterus	Non-endometrioid	G3	Pelvis	0	0	0
25	M	59	Kidney	Clear cell	G3	Fibula	0	0	0

LF, lactoferrin; ASP, area of staining positivity; IS, intensity of staining; ID, intensity distribution.
